# Nanocomposite Zinc Oxide-Chitosan Coatings on Polyethylene Films for Extending Storage Life of Okra (*Abelmoschus esculentus*)

**DOI:** 10.3390/nano8070479

**Published:** 2018-06-29

**Authors:** Laila Al-Naamani, Joydeep Dutta, Sergey Dobretsov

**Affiliations:** 1 Department of Marine Science and Fisheries, Sultan Qaboos University, PO Box 34, Al-Khoud, 123 Muscat, Oman; Lnaamani@hotmail.com; 2 Ministry of Municipalities and Water Resources, 112 Muscat, Oman; 3 Functional Materials, Applied Physics Department, SCI School, KTH Royal Institute of Technology, Kista, SE-164 40 Stockholm, Sweden; joydeep@kth.se; 4 Center of Excellence in Marine Biotechnology, Sultan Qaboos University, PO Box 50, Al-Khoud, 123 Muscat, Oman

**Keywords:** ZnO nanoparticle, nanocomposite coating, chitosan, antimicrobial, active food packaging

## Abstract

Efficiency of nanocomposite zinc oxide-chitosan antimicrobial polyethylene packaging films for the preservation of quality of vegetables was studied using okra *Abelmoschus esculentus*. Low density polyethylene films (LDPE) coated with chitosan-ZnO nanocomposites were used for packaging of okra samples stored at room temperature (25 °C). Compared to the control sample (no coating), the total bacterial concentrations in the case of chitosan and nanocomposite coatings were reduced by 53% and 63%, respectively. The nanocomposite coating showed a 2-fold reduction in total fungal concentrations in comparison to the chitosan treated samples. Results demonstrate the effectiveness of the nanocomposite coatings for the reduction of fungal and bacterial growth in the okra samples after 12 storage days. The nanocomposite coatings did not affect the quality attributes of the okra, such as pH, total soluble solids, moisture content, and weight loss. This work demonstrates that the chitosan-ZnO nanocomposite coatings not only maintains the quality of the packed okra but also retards microbial and fungal growth. Thus, chitosan-ZnO nanocomposite coating can be used as a potential coating material for active food packaging applications.

## 1. Introduction

Fruits and vegetables being perishable due to high water content are susceptible to rapid deterioration soon after harvest, requiring them to be properly packaged and stored if not consumed immediately [1]. High perishability of most fruits and vegetables has led investigators to seek new approaches to improve shelf-life. Okra (*Abelmoschus esculentus*, family Malvaceae) is a rich source of vitamin C, calcium, carotene, vitamin B1, folates and contains dietary fibre [2,3] and is a widely grown and consumed vegetable in African and Arabic countries [4]. However, it has a short postharvest life of about 10 days at temperatures from 1 to 10 °C due to high respiration and water loss rates [5,6]. It is quite sensitive to bruising, desiccation, loss of chlorophyll, chilling injury, loss of tenderness (increased toughness), that usually leads to decaying following postharvest handling [5].

Packaging plays a critical role in food safety and quality acting as a barrier that protects the food from the outer environmental conditions, such as contamination by pathogens and spoilage organisms, chemical and physical hazards, thus slowing the process of deterioration [7,8]. Food packaging with antimicrobial properties has received attention due to the ability to arrest or delay microbiological decay of food products [9]. In antimicrobial packaging materials, antimicrobial substances are loaded in the packaging system to reduce the risk of contamination by pathogens [10,11]. One of the most successful approaches of antimicrobial packaging development is the coating of polymer surfaces with antimicrobial substances. As bacterial contamination occurs on the surface, the incorporation of antimicrobial agents as a coating provides high exposure area with minimal dissociation of biocides into the packaged food [12,13]. Recent studies have demonstrated that antimicrobial packaging improves the safety and quality of food products and helps in reducing the amount of preservatives in the food [14]. The preservative action of antimicrobial packaging is based on the release of the agents from the packaging material, which do the preservative action by direct contact with the food or by releasing to the surrounding space [14].

Natural polymers, such as chitosan, starch, clay, and pectin, have been used in food packaging due to their biodegradability, non-toxicity and biological properties [15,16,17]. Chitosan is non-toxic, film forming, and biodegradable biopolymer with antimicrobial properties, which make it ideal for the development of antimicrobial food packaging [17,18]. It is widely used in food production as a fining agent for clarification and de-acidification of fruit juices and for water purification [19]. Chitosan as a packaging material either alone or with other compounds was proved to improve shelf life while maintaining the quality of meat [20], fish [21] and vegetables [17]. Chitosan can form semipermeable film on fruits and vegetables, introducing host resistance to pathogens [22]. Delay in ripening and shelf life prolongation was observed in fruits and vegetables treated with chitosan [23]. Chitosan is usually blended with other polymers or antimicrobial agents, such as natural extracts and metal oxides to improve its mechanical resistance and antimicrobial properties [24,25]. For example, chitosan was blended with other active substances, such as gallic acid [26], grapefruit seed extract [27], poly-vinyl alcohol [17] and silver nanoparticles [28] to form effective antimicrobial packaging materials. 

Metal oxides like titanium dioxide (TiO_2_), zinc oxide (ZnO) and magnesium oxide (MgO), have been reported to render antibacterial activity with higher stability in comparison to organic antimicrobial agents [29,30]. In comparison to other metal oxides, ZnO nanoparticles are considered as safe materials for human beings [31]. Zinc is a ubiquitous trace metal and essential for a large number of metalloenzymes in living organisms [7]. Furthermore, ZnO is less toxic than other nanoparticles, such as silver nanoparticles, thus is widely used in the food industry as a supplement for zinc [7,32]. ZnO has been incorporated into the linings of food cans for meat, fish, corn and peas to preserve colour and prevent spoilage [32]. Interest has been arisen on using ZnO nanoparticles as food additives or incorporating them with packaging materials in order to provide antimicrobial properties [7,33,34]. Several studies proposed methods used to incorporate ZnO nanoparticles with low density polyethylene (LDPE), polypropylene (PP) and chitosan [32]. The application of chitosan coating in food was mostly presented as an edible or direct coating. Few reports proposed the use of chitosan films blended with different components for food packaging [17,18,19,20,21]. A recent study by Rahman and co-workers [20] proved the efficiency of chitosan-ZnO nanocomposite films formed into pouches in extending the shelf-life of raw meat. However, to the best of our knowledge, there are no reports on the use of chitosan-ZnO coated packaging materials and their application in food preservation.

In this study, the effect of low density polyethylene (LDPE) packaging films coated with chitosan and chitosan-ZnO nanocomposite on the shelf life and quality of okra (*Abelmoschus esculentus*) was investigated. The specific aims of this study were to: (1) prepare LDPE packaging coated with chitosan and chitosan-ZnO nanocomposites; (2) assess the quality of okra samples stored using active packaging through the evaluation of the microbiological, chemical and physical attributes of the vegetable.

## 2. Materials and Methods

### 2.1. Sample Preparation

**Figure 1:**
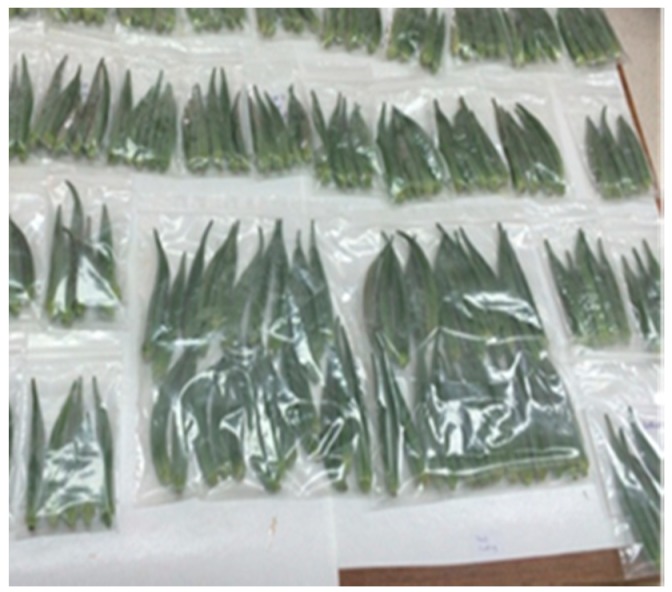
Photo of all the Okra samples in different polyethylene packages.

Fresh okra (*Abelmoschus esculentus*) samples were purchased from a local supermarket (As Seeb, Muscat, Oman). Samples were transported to the marine research laboratory and then they were graded visually for their uniformity in size, shape and brightness of colour. Pieces with average size of 13 cm × 2 cm (length × width) were selected for the experiment (Figure 1). Only vegetables free from insects, defects and visible blemishes were selected and used for the experiments (see below).

### 2.2. Preparation and Characterisation of Coatings

LDPE films coated with chitosan and chitosan-ZnO nanocomposites were prepared as previously described by Al-Naamani and co-workers [35]. Briefly, 2% chitosan solution was prepared by dissolving 2 g of chitosan powder (Sigma Aldrich, St. Louis, MO, USA) in 0.5% acetic acid. Then, commercial ZnO nanoparticles (35–45 nm) (Sigma Aldrich, St. Louis, MO, USA) were added to the previously prepared chitosan solution to obtain chitosan-ZnO nanocomposite. Clean LDPE films (5 × 8 × 2 cm) (Cole-Parmer Instrument Co., Cambridgeshire, UK) were treated using plasma instrument (Plasma Technology GmbH, Herrenberg-Gültstein, Germany) (pressure: 0.2 mbar, O_2_: 3–4 standard cubic centimetres per minute (SCCM), power: 50%). The atmospheric dielectric barrier discharge (DBD) plasma operated at 22 kHz eliminating the need for impedance matching that is required for inductively coupled (ICP) plasma, radio frequency low pressure (RFP) plasma systems, making it simpler to use for a variety of applications including ashing of organic constituents, cleaning of electron microscopy sample holders and all sample surfaces, etching or structuring of surfaces as well as for the modification of surface properties (hydrophilic/hydrophobic). The system uses 230 V/16 A power consumption including vacuum pump (approximately 800–1200 W) (Pfieffer, Annecy, France) with power that can be chosen from 10% to 100%. Plasma treatment was used to provide a hydrophilic property to the polyethylene (PE) surface in order to result in a better attachment of chitosan to the PE surface. Oxygen or air plasma is known to removes organic contaminants by chemical reaction with highly reactive oxygen radicals and through ablation by energetic oxygen ions, promotes surface oxidation and hydroxylation (OH groups) thus increasing surface wettability. After the plasma treatment, 6 mL of previously prepared chitosan-ZnO nanocomposite solution was sprayed onto the PE surface (10 cm × 15 cm) and allowed to dry at room temperature (26 °C). PE films coated with 2% chitosan were used for comparison, and uncoated PE was used as a control. The coated films were characterized and their antimicrobial activity was reported [35]. 

Surface morphology of the nano-composite coating was characterized by JEOL JSM-7200 (JEOL Ltd., Akishima, Tokyo, Japan) field emission scanning electron microscope (FESEM) working at 20 kV. The elemental composition of the coatings was determined using Energy Dispersive Spectrometry (EDS) (Oxford Instruments NanoAnalysis & Asylum Research, UK, High Wycombe, UK). The static water contact angles of the nano-composite coatings were measured using a Theta Lite attension tensiometer (Biolin Scientific, Gothenburg, Sweden) using sessile drop technique in order to determine film hydrophobicity. Fourier Transform Infra-red (FTIR) spectroscopy was used to identify the chemical structure of the composite films and the possible interactions between their components. The coated and uncoated LDPE films were analysed using Attenuated Total Reflection (ATR) attachment in Frontier (FTIR) spectrometer (PerkinElmer, Waltham, MA, USA), in a spectral range from 4000 to 500 cm^−1^. 

For determination of Zn ions concentration leached per cm^2^ of coated LDPE, pieces of the coated LDPE (5.5 cm × 2.5 cm) were immersed in 20 mL distilled water and kept under agitation (100 rpm) for the duration of the experiment. Water samples were collected and measured using Inductively Coupled Plasma Optical Emission Spectroscopy (ICP-OES) (Varian 710 ES, Santa Clara, CA, USA) for Zn determination. The analysis was done in 6 replicates.

### 2.3. Experimental Design

Selected okra samples (see sample preparation) were separated into three equal groups for the study. First group was packed in the LDPE films coated with chitosan. The second group was packed in LDPE coated with chitosan-ZnO nanocomposite. The last group was used as a control and the samples were packed in uncoated LDPE films. All okra samples were then stored for 12 days at room temperature (25 °C) for subsequent quality assessment (Figure 1). To determine the overall quality of the samples, analysis of the chemical, physical and microbiological counts of all okra samples (see below) were carried out upon 4 and 8 and 12 days of storage.

### 2.4. Microbial Analysis

Total bacteria and total fungal count in packed okra samples was determined according to the method reported by Harrigan [36]. Briefly, 10 g of each samples were aseptically homogenized with 90 ml of peptone water for 1 min. Ten-fold serial dilution of homogenate samples were prepared and 0.1 mL of each of these solutions were added onto nutrient agar (Sigma Aldrich, St. Louis, MO, USA) for bacterial analysis or potato dextrose agar (Sigma Aldrich, St. Louis, MO, USA) for fungal analysis. The inoculated agar plates were incubated at 37 °C for 24 h for bacterial counts. The Petri dishes prepared for fungal counts were incubated at 30 °C for 5 days. Samples were prepared in triplicate, and the counts between 30 and 300 CFU/g were only considered. All the results are expressed as log_10_ CFU/g (CFU—colony forming units).

### 2.5. Chemical and Physical Properties of Okra Samples

For the determination of pH, 5 g of okra sample after each treatment was homogenized thoroughly with 45 mL of distilled water using a blender (Panasonic Corporation, Tokyo, Japan) whereupon the pH was determined using a pH meter (WTW, Weilheim, Germany). Total soluble solids concentration was measured with a pocket refractometer (ATAGO, Tokyo, Japan). In order to do this, the samples were first homogenized in a blender (Panasonic Corporation, Tokyo, Japan). Then, homogenised samples were placed on the prism glass of the refractometer to take direct readings. Moisture content (wet basis) was calculated from the change in sample weight (initial weight–final weight) determined using a balance (Sartorius, Goettingen, Germany) after drying the samples in a vacuum oven at 70 °C for 24 h. Okra weight loss was calculated by weighing okra samples before and at the last day of storage. The results, represented as means ± standard deviation of measurements, were obtained from 10 randomly chosen samples per treatment. The difference between the initial and the final weight of the samples was considered as a total weight loss. The results are expressed as percentage loss of the initial weight.

### 2.6. Statistical Analysis

Data were subjected to the analysis of variance (ANOVA). Before the analysis, assumption of normality of the data was verified using the Shapiro-Wilk test [37]. Source of variation were the different treatments of the packaging film and storage time. Significant difference between different measurements were determined by Fisher Least Significant Difference (LSD) post hoc test, at a significant level *p* = 0.05. 

## 3. Results

### 3.1. Characterisation of Coated PE Films

**Figure 2:**
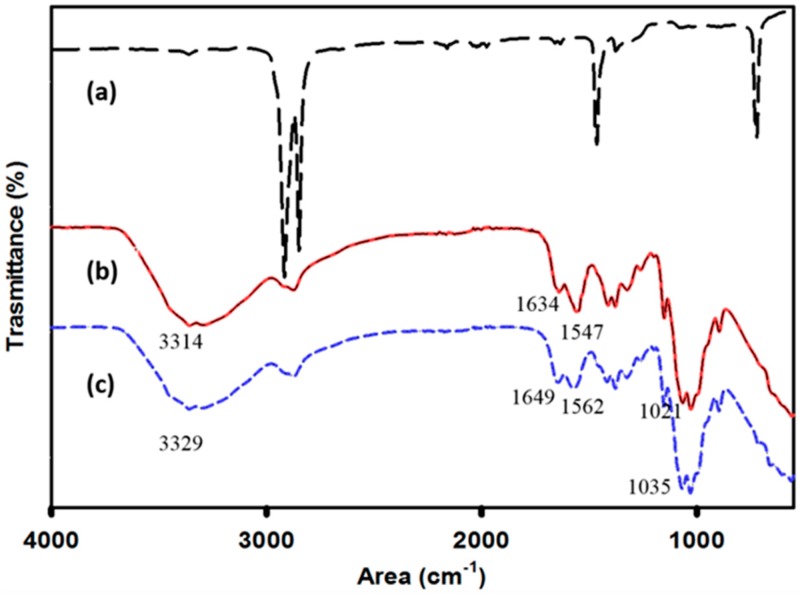
Fourier transform infrared FTIR spectra of low density polyethylene films (LDPE) films coated with chitosan and chitosan-ZnO nanocomposite compared to uncoated LDPE: (**a**) uncoated LDPE; (**b**) LDPE coated with chitosan-ZnO nanocomposite; (**c**) LDPE coated with chitosan.

The FTIR spectra of uncoated LDPE, and LDPE coated with chitosan and chitosan-ZnO nanocomposite are shown in Figure 2. The LDPE spectrum observed in this study was similar to one reported previously [38]. Methylene (CH_2_) groups corresponding to the stretching modes at 2920 and 2850 cm^–1^ and deformations at 1464 and 719 cm^–1^ as shown in Figure 2 are well known in PE. After the plasma treatment, new peaks at 1720 cm^–1^ corresponding to C=O stretching vibration and at the region of 3200–3800 cm^–1^ corresponding to hydroxyl group (–OH) vibration can be usually observed. For the chitosan coated PE films, the characteristic peaks of chitosan were observed at 3329 cm^−1^ (N–H and O–H stretching) and at 1649 and 1562 cm^−1^ (amide I and amide II) (Figure 2c). A peak at 1035 cm^−1^ was attributed to the stretching vibration of C–O–C [39], which suggests that chitosan is chemically bonded to polyethylene. In comparison, the spectrum of chitosan-ZnO nanocomposite coating has a slight shift of the bands corresponding to hydroxyl, amino, and amide groups towards lower spectral ranges (Figure 2b). This is attributed to the interaction between chitosan and ZnO nanoparticles.

**Figure 3:**
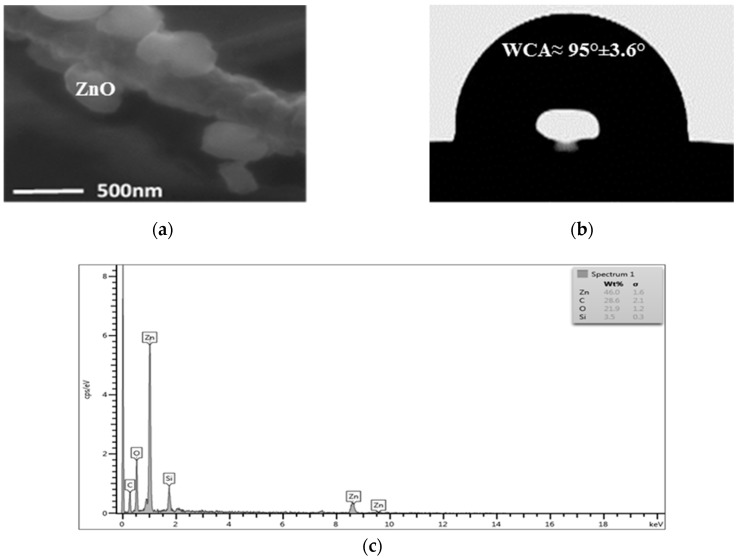
Characterisation of LDPE films coated with chitosan-ZnO nanocomposite. (**a**) SEM image of the coated LDPE (20,000 ×); (**b**) Measurement of static contact angle of a water droplet on coated LDPE, the data are means ± standard deviations of five replicates; (**c**) Energy Dispersive Spectrometry (EDS) spectrum of LDPE surface coated with Chitosan-ZnO nanocomposite. Each peak represents different elements.

Scanning Electron micrographs (SEM) confirmed the agglomeration of ZnO nanoparticles to about 500 nm in the nanocomposite coating (Figure 3a). The nanocomposite coating exhibits a hydrophobic surface with a water contact angle of ~95° (Figure 3b). The EDS profile proves that ZnO nanoparticles were successfully incorporated into the chitosan matrix as peaks of zinc were shown in the spectra (Figure 3c). ICP analysis demonstrated that total Zn^2+^ ion concentration that leached out from the nanocomposite coating was 0.00147 ± 0.00008 mg/cm^2^ when it was kept under agitation for the duration of the experiment.

### 3.2. Microbial Analysis of Packed Okra

**Figure 4:**
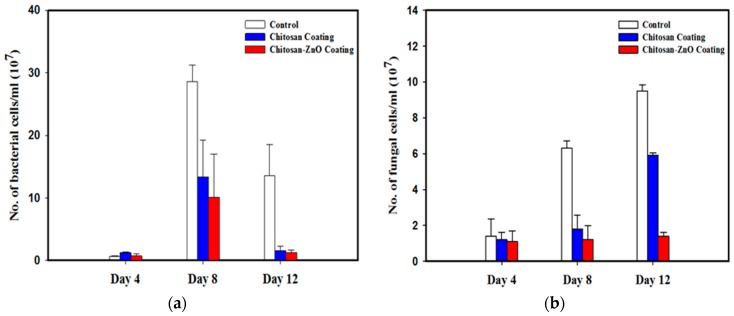
Number of (**a**) bacterial and (**b**) fungal cells (CFU/ml) in okra samples packed in coated and uncoated LDPE films, and incubated for 4, 8 and 12 days.

The bacterial concentration in packed okra samples varied during the whole storage period (Figure 4a). In the first 4 days of storage, there was no significant difference (ANOVA, LSD, *p* > 0.05) in the bacterial concentration between the control (LDPE films) or either of the treated PE films (Figure 4a). After 8 days, the bacterial CFUs increased dramatically in all the samples. Compared to the control sample, the bacterial concentrations in the case of chitosan and nanocomposite coatings were reduced by 53% and 63% respectively, though there was no significant difference (ANOVA, LSD, *p* > 0.05) between the treatments. At the end of experiment, the concentrations of bacteria in all the samples decreased. Similar to day 8, both coatings lead to the reduction of bacterial counts in okra compared to the control (Figure 4a). There was no significant difference (ANOVA, LSD, *p* > 0.05) between the bacterial concentrations in the treated samples.

The fungal concentrations did not differ significantly (ANOVA, LSD, *p* > 0.05) in the first 4 days of storage in all the samples (Figure 4b). Similar to the bacterial counts, fungal concentrations in the samples stored with chitosan and nanocomposite coated films were significantly different (ANOVA, LSD, *p* < 0.05) from the control experiments upon 8 and 12 days of storage. There was no significant difference (ANOVA, LSD, *p* > 0.05) between the fungal concentrations in the chitosan and the nanocomposite samples after 4 and 8 days, but after 12 days fungal counts in samples stored in nanocomposite coated films decreased more than 2-folds (ANOVA, LSD, *p* < 0.05) in comparison to the samples stored in chitosan coated LDPE films. In the control samples the fungal concentrations increased incrementally with storage time (Figure 4b). 

### 3.3. Chemical and Physical Properties of Packed Okra

The pH values of okra samples packed with uncoated LDPE, chitosan coated LDPE and LDPE coated with chitosan/ZnO nanocomposite films are presented in Table 1. There was a slight increase in acidity of all of packed okra samples during the experiments. After 12 days of storage, pH values decreased by 1.08%, 2.6%, 1.85% in samples packed in control LDPE films or chitosan and nanocomposite coated films, respectively with no significant difference (ANOVA, LSD, *p* > 0.05).

The amount of total soluble solids in the okra samples increased during the experiment over the period of storage (Table 2). After 4 days of storage, the amount of total soluble solids in the control samples were significantly lower (ANOVA, LSD, *p* < 0.05) than that in the samples stored in either chitosan or nanocomposite coated LDPE films (Table 2). However, all the values increased in the eighth day with no significant difference (ANOVA, LSD, *p* > 0.05) between them. After 12 days, the amount of total soluble solids in the sample with chitosan-ZnO nanocomposite coating was significantly lower (ANOVA, LSD, *p* < 0.05) than the control (Table 2).

Moisture content of okra stored at room temperature in the three different packaging materials is shown in Table 3. The moisture content in all samples decreased gradually during the experiment. In the first 4 days of storage, moisture content decreased insignificantly (ANOVA, LSD, *p* > 0.05) by 2.3%, 1.99% and 1.6% for the okra stored in LDPE (control) film or chitosan and nanocomposite coated films, respectively. After 8 days, the control sample lost 10-fold more moisture (ANOVA, LSD, *p* < 0.05) than the samples packed stored in coated packages (Table 3). The control sample lost most of the moisture content within the first 8 days of storage. After 12 days okra samples lost 3.4%, 2.9%, and 2.6% of total moisture from the samples stored in control LDPE films or chitosan and nanocomposite coated films, respectively. The difference between the treatments and the control were not significant.

Insignificant variation (ANOVA, LSD, *p* > 0.05) in weight loss was observed among okra samples packed in the two coated films and the control after 12 days of storage. Samples exhibit values of 2.17% ± 0.62, 2.32% ± 1.91 and 2.03% ± 1.24 of weight loss when stored in uncoated films, chitosan or nanocomposite coated LDPE films, respectively.

## 4. Discussion

The efficiency of any antimicrobial packaging material can be evaluated by several factors. Ideal packaging should provide minimal dissociation of the incorporated antimicrobial agent into the packaged food as well as improve food safety by retarding microbial growth without changing its quality attributes [12,13,14]. In our experiment, chitosan was incorporated with ZnO nanoparticles and coated onto polyethylene films in order to develop antimicrobial packaging for food storage. This fabricated packaging was characterised and tested for its efficiency in increasing shelf life of okra samples.

The dissociation of Zn^2+^ ions from the packaging coating was determined throughout the experiment period. The concentration of zinc in the coating was 0.08 mg/cm^2^. After 12 days, the percentage of Zn^2+^ ions released was calculated to be about 1.8% of the total amount of zinc in the coating. These results showed that zinc release from the fabricated packaging material was still very low even though force was applied to intentionally increase release of Zn^2+^ from the coating. This confirms the stability of ZnO nanoparticles in the chitosan matrix. Chitosan was reported to form a network with ZnO nanoparticles when blended together, which could control the release of Zn^2+^ ions into the environment [40]. Zinc concentration value reported in our study was much lower than the lethal dose concentration estimated for humans on comparison with equivalent studies in animals [41]. As reported by SCF [42], the recommended upper intake level of Zn for human is 25 mg/day.

Antimicrobial activity is a very important factor to evaluate the efficiency of the packaging to be used for food storage. The coatings used in our experiment showed good antimicrobial activity by reducing the amount of bacterial and fungal growth in packed okra samples during the twelve days of storage. In previous studies, the antimicrobial effect of direct chitosan coating of fruits and vegetables has been reported [19,43,44,45]. Chitosan reduced growth of grey mould [46,47], blue mould [48] and black mould [49] in grapes, strawberries and tomatoes. The growth of foodborne bacteria, such as *E. coli* in tomato [44] and *Salmonella* spp. in whole cantaloupe [45], was reported to get controlled upon the use of chitosan. Antimicrobial effect of plastic bags coated with ZnO nanoparticles was reported previously. Li and co-workers [50] reported 30% reduction in *E. coli* count in cut apple stored in ZnO coated polyvinyl chloride (PVC) bags. Similarly, it was reported by Emamifar and co-workers [51] that the application of low density polyethylene (LDPE) packages blended with ZnO nanoparticles reduced total aerobic bacteria and total yeast and mould in fresh orange juice as well as prolonged its shelf life up to 28 days at 4 °C without any negative effects on sensory quality of the juice. In our study ZnO nanoparticles were mixed with chitosan and bags were coated. Since ZnO nanoparticles and chitosan have antimicrobial properties, we expected a synergistic effect. At the same time, the synergistic effect was observed only on reduction of fungal growth in the okra samples but no significant variation could be found in the inhibition of bacterial growth. Malini and co-workers [52] reported strong antibacterial activity of the chitosan-ZnO nanocomposite membrane with higher inhibition of the Gram negative *K. planticola* than Gram positive *Bacillus subtilis*. Liu and Kim [53] reported a similar synergistic effect of chitosan and ZnO nanoparticles against bacteria *E. coli*, *P. aeruginosa*, *S. aureus*, and *B. subtilis*. A study by Rahman and co-workers [20] revealed that raw beef meat packed into pouches made of chitosan films incorporated with 2% ZnO nanoparticles showed complete inhibition of bacterial growth on the sixth day of storage at 4 C. The ZnO concentration used in this study was 20 times higher than the concentration used in our study (0.1%), which can explain the obtained results. Several mechanisms have been proposed for the strong antimicrobial and antifungal activities of chitosan, most of them owing to the interaction of positive charges of chitosan with the negative charges of microorganisms’ cells membrane [54]. It was reported that chitosan can have an effect on the permeabilization of cell membranes of some fungal species depending on their membrane fluidity [19]. Antifungal effect of chitosan can be caused by the biological mechanisms, such as inducing morphological changes, structural alterations of the fungal cells and fruit resistance induction to pathogen attacks [55]. The antimicrobial activity of ZnO nanoparticles is probably related to the photocatalytic generation of reactive oxygen species (ROS) on the surface of the nanoparticles [56,57,58]. ROS and Zn^2+^ ions are supposed to interact with the anionic components of microbial cell wall causing leakage of these components leading finally to cell death [59]. In the case of ZnO-nanocomposite coatings, synergistic activity was probably due to the enhancement of the positive charges of the amino group of chitosan by ZnO which lead to stronger interaction with negatively charged microbial cell wall [20].

Assessment of quality attributes of packed okra samples was done by measuring pH, total soluble solids, moisture contents and weight loss of samples after each storage time. These measurements give indication of efficiency of the fabricated packaging in preserving quality characteristics of okra samples. The results showed that while acidity of okra samples slightly increased during the twelve days of storage, the pH values did not show any significant changes (ANOVA, LSD, *p* > 0.05). Babarinde and Fabunmi [1] had earlier reported a reduction in pH from 6.7 to 6 of okra samples stored in LDPE films for 9 days. However, there were no other reports, either on okra coated with chitosan or stored in chitosan coated films. Similarly, the effect of storage of okra in chitosan-ZnO nanocomposite coated LDPE films has not been investigated. However, an increase in the pH of grapes honey melon was observed when directly coated with chitosan [19]. In comparison, Hernández-Muñoz and co-workers [60] observed a reduction in pH of strawberries coated with chitosan. These differences in the results about the change in acidity of fruits and vegetables could be attributed to their respective organic contents [19].

Total soluble solids indicate the proportion of dissolved solids, such as sugars, acids, amino acids, ascorbic acids and minerals in fruits and vegetables [61,62]. It is a refractometric index usually measured in Brix units which are equal to per cent of soluble solids. The increase in concentration of the total soluble solids in the sample is directly related to the increase in water loss during the storage period. The minimal effect of the chitosan coating in total soluble solids in our experiment could be due to the use of indirect coating on the package films instead of direct coating on food itself. It was reported that retention of total soluble solids in chitosan coated fruits, such as pears [63], strawberries [60] and banana [64] improved. Direct coating on fruits was reported to reduce respiration level which slows down synthesis and use of metabolites leading to slower carbohydrate hydrolysis into sugars leading to a reduction of the concentration of soluble solids [65,66]. However, this process depends on different other factors such as coating thickness, storage conditions and type of fruit and its ripeness stage [65].

Moisture loss in vegetables occurs due to the post-harvest physiological processes, such as respiration and transpiration [1]. The low water loss in all the samples in our experiment could be arise due to the good barrier properties of LDPE to water vapour loss and the ability to reduce respiration rate of vegetables [67,68,69]. It was reported previously that initial moisture content of okra is 88% and it dropped to 85% after 9 days storage in LDPE [1]. This rate is higher than moisture reduction rate observed in our experiment, which could be attributed to differences in the barrier properties of LDPE films used.

Packaging films is known to lead to the establishment of high relative humidity inside the package due to the reduction in water diffusion to the atmosphere. Thus, transpiration rate is reduced which in turn decrease weight loss in okra [70]. Babarinde and Fabunmi [1] reported 5.8% weight loss in okra after 9 days storage in LDPE when it was stored at 28 °C. The lower loss in moisture (3.9%) was observed when the samples were stored at lower temperatures (15 °C). Difference between this and our results probably due to differences in okra species and packaging films used in our study. Since there were no reports in okra packed LDPE coated with chitosan or chitosan/ZnO nanocomposite, it was not possible to compare the obtained results directly.

From all the results obtained in this work on properties of packed okra upon prolonged storage, it can be observed that the coatings on LDPE package did not influence the chemical and physical properties of okra, such as pH, total soluble solids and moisture content. As mentioned earlier, this could be attributed to the use of indirect coating of chitosan on the plastic film. Direct coating of chitosan on fruits and vegetable are reported to affect the physical and chemical properties of coated grapes, apple, pear, tomato, sweet pepper amongst others [49,71,72,73,74]. Chitosan was reported to reduce oxygen and elevating carbon dioxide levels in coated fruits by providing a semipermeable film around them. This can modify internal atmosphere decreasing the respiration level and metabolic activities of fruits and results in ripening delays [23,75,76]. Manipulation of respiration levels can influence properties such as total soluble solids, moisture, weight loss and pH of fruits and vegetables [23]. For example, because of chitosan semipermeable barrier and its reduction of respiration rate, reduced pH and weight loss of coated apples [74] and reduced weight loss of pears [63,73] were reported.

## 5. Conclusions

This study showed an improvement in the performance of coated LDPE films with chitosan and with chitosan/ZnO nanocomposites for the preservation of quality of okra samples by maintaining moisture content, total soluble solid and pH as well as preventing bacterial and fungal growth in the stored okra samples. Okra pods harvested with minimum handling were reported to have minimum rotting (3.0%) and good appearance for the first 5 days that could be extended upon cold storage for less than 2 weeks [77]. The obtained results proved the effectiveness of the nanocomposite coating on the reduction of fungal growth in the okra samples for up to 12 storage days. Significant reduction in bacterial growth was observed in the samples stored in treated polyethylene films compared to the control, and the nanocomposite coating performed better for the prevention of fungal growth than chitosan alone. It can be concluded that LDPE coating with chitosan-ZnO nanocomposite is a promising technique in which antimicrobial property is added to the films which could influence its possible applications as active food packaging to prolong shelf-life of packed food.

## Figures and Tables

**Table 1:** Effect of different coatings on pH of okra samples at each storage periods.

Coating Material	Storage Duration (Days)			
	0	4	8	12
Control (uncoated LDPE)	6.47 ± 0.06 ^a,x^	6.44 ± 0.05 ^a,x^	6.40 ± 0.07 ^a^	6.40 ± 0.01 ^a^
Chitosan coating	6.47 ± 0.06 ^a,x^	6.43 ± 0.03 ^a,x^	6.30 ± 0.06 ^b,x^	6.30 ± 0.07 ^b,x^
Chitosan/ZnO coating	6.47 ± 0.06 ^a,x^	6.34 ± 0.02	6.29 ± 0.07 ^b,x^	6.35 ± 0.03 ^ab,x^

Note: ± = standard deviation; Values followed by the same letters in a column (a,b) or in a row (x,y) do not differ significantly.

**Table 2:** Effect of different coatings on total soluble solids (brix) in okra samples during different storage periods.

Coating Material	Storage Duration (Days)			
	0	4	8	12
Control (uncoated LDPE)	4.7 ± 0.8 ^a,x^	3.6 ± 0.5 ^b,x^	5.5 ± 1.0 ^a,x^	7.0 ± 0.6 ^a^
Chitosan coating	4.7 ± 0.8 ^a,x^	4.8 ± 0.9 ^a,y^	4.9 ± 0.4 ^a,x^	6.0 ± 0.5 ^ab^
Chitosan/ZnO coating	4.7 ± 0.8 ^a,y^	5.3 ± 0.4 ^a,x^	5.3 ± 0.2 ^a,y^	5.5 ± 0.1 ^b^

Note: ± = standard deviation; Values followed by the same letters in a column (a,b) or in a row (x,y) do not differ significantly.

**Table 3:** Effect of different coatings on moisture content (%) of okra samples during different storage periods.

Coating Material	Storage Duration (Days)			
	0	4	8	12
Control (uncoated LDPE)	86.3 ± 1.1	84.2 ± 0.4	83.4 ± 1.1	83.4 ± 1.6
Chitosan coating	86.3 ± 1.1	84.6 ± 0.7	84.1 ± 0.9	83.3 ± 0.8
Chitosan/ZnO coating	86.3 ± 1.1	85.2 ± 0.9	84.9 ± 0.6	84.0 ± 1.0
